# Pathological respiratory chemoreflex activation predicts improvement of neurocognitive function in response to continuous positive airway pressure therapy

**DOI:** 10.3389/fnins.2025.1619467

**Published:** 2025-09-24

**Authors:** Yu-Tong Hu, Yue-Nan Ni, Hugi Hilmisson, Robert Joseph Thomas

**Affiliations:** ^1^Department of Respiratory, Critical Care and Sleep Medicine, West China School of Medicine and West China Hospital, Sichuan University, Chengdu, China; ^2^Department of Respiratory Care, West China School of Medicine and West China Hospital, Sichuan University, Chengdu, China; ^3^MyCardio-LLC, Denver, CO, United States; ^4^Division of Pulmonary, Critical Care and Sleep Medicine, Beth Israel Deaconess Medical Center, Boston, MA, United States

**Keywords:** sleep apnea, positive airway pressure, cardiopulmonary coupling, neurocognitive function, high loop gain

## Abstract

**Introduction:**

There is a need for biomarkers predicting neurocognitive improvement following treatment of obstructive sleep apnea (OSA) with continuous positive airway pressure (CPAP). The role of sleep apnea endotypes as predictors are promising.

**Objective:**

To assess the relationship between a high loop gain biomarker, elevated low frequency narrow band (e-LFC_NB_), and improvements in neurocognitive function in the Apnea Positive Pressure Long-term Efficacy Study (APPLES).

**Methods:**

The e-LFC_NB_ % metric was estimated on baseline polysomnography. Logistic regression analysis was performed to identify the potential association between e-LFC_NB_% of total sleep time and the observed improvement in neurocognitive function following the specified treatment.

**Results:**

A total of 362 subjects received CPAP and had e-LFC_NB_ % measurements. For Sustained Working Memory Test-Overall Mid-Day (SWMT-OMD), e-LFC_NB_% > 2.35% correlates positively with the proportion of participants who showed an increase in test scores > 0.65 after 2 months CPAP treatment (OR: 2.617, 95% CI: 1.095–6.252, *p*: 0.030); e-LFC_NB_% > 9.45% correlates positively with improvement in test scores > 0.8 after 6 months CPAP treatment (OR: 2.553, 95% CI: 1.017–6.409, *p*: 0.046). For Buschke Selective Reminding Test sum recall (BSRT-SR), e-LFC_NB_% > 3.65% correlates positively with an increase in test scores > 12 after 2 months CPAP treatment (OR: 2.696, 95% CI: 1.041–6.982, *p*: 0.041). Results of the Pathfinder Number Test-Total Time (PFN-TOTL) were not significant.

**Conclusion:**

e-LFC_NB_% (probable high loop gain) may be a clinically useful predictor of cognitive improvement following CPAP.

## Background

Obstructive Sleep Apnea (OSA) is a sleep-related respiratory disorder characterized by recurrent collapse of the upper airway during sleep (McNicholas and Pevernagie, 2022). This condition often leads to intermittent hypoxia, cytokine activation, dysmetabolism, nocturnal blood pressure surges, and sleep fragmentation (Kapur et al., 2017), which are commonly considered contributors to neurocognitive dysfunction associated with OSA (He et al., 2024; Vanek et al., 2020). There is an extensive body of research indicating that OSA is associated with a broad range of neurocognitive impairments, with the most prevalent reports focusing on memory and new learning, as well as attention and executive function (Bucks et al., 2013).

Continuous positive airway pressure (CPAP), which is regarded as the first line therapy for OSA, can improve hypoxia in OSA patients, reduce sleep fragmentation, and reverse sympathetic nerve excitation (Patil et al., 2019), but there is controversy regarding whether CPAP can improve cognitive dysfunction caused by OSA (Bucks et al., 2013). The existence of complex pathologies especially high loop gain maybe a reason for the failure due to impairment in both effectiveness and adherence (Ni and Thomas, 2023; Cheng et al., 2024). It is also plausible that pathological respiratory chemoreflex activation and the resulting heightened sympathetic activation and other pleotrophic effects such as oxidative stress, inflammatory response, and neuronal damage (Lv et al., 2023) may influence development of neurocognitive dysfunction, and response to treatment.

High loop gain within the respiratory system, a hallmark of unstable respiratory control, typically occurs during non-rapid eye movement sleep (NREM) (Deacon and Catcheside, 2015). Research indicates that in patients with OSA, respiratory control is significantly less stable during NREM sleep compared to rapid eye movement sleep (REM). This instability may contribute to cognitive decline via enhancing sleep fragmentation (Joosten et al., 2021). Additionally, high loop gain is a marker of carotid body activation, which is often linked to increased sympathetic nerve drive and glucose dysmetabolism (Joyner et al., 2018; Sacramento et al., 2020). That nighttime sympathetic activation may be an important factor in impairing the neurocognitive functions of patients with OSA has been reported (Alomri et al., 2021).

As described in our previous work, narrow band elevated low frequency coupling (e-LFC_NB_) measured by cardiopulmonary coupling sleep spectrograms is a biomarker indicating periodic breathing and central sleep apnea (Thomas et al., 2007), indicating high loop gain. Our previous study showed that e-LFC_NB_ was a strong predictor for blood pressure dropping after CPAP in OSA (Ni et al., 2024). However, whether it influences neurocognitive function improvement by CPAP is not known.

This study conducted a secondary analysis of the Apnea Positive Pressure Long-term Efficacy Study (APPLES). Our hypothesis was that e-LFC_NB_%, as a marker of unstable respiratory control and a sign of carotid body activation, could predict neurocognitive change with CPAP.

## Method

### Study design

This study utilized data from the Apnea Long-Term Efficacy Study (APPLES), a multicenter, randomized, double-blind trial with a two-arm, sham-controlled design aimed at assessing the impact of CPAP compared to sham CPAP on cognitive function. A secondary analysis was performed following approval from the Institutional Review Board, leveraging data available through the National Sleep Research Resource (NSRR) at: www.sleepdata.org. The study’s methodology and primary findings have been thoroughly documented in a prior publication (Kushida et al., 2012).

### CPAP treatment

Participants assigned to the CPAP group underwent an overnight sleep laboratory study to determine the optimal therapeutic pressure. They were equipped with a REMstar Pro CPAP system featuring heated humidification, along with the necessary mask and tubing. Meanwhile, those in the control group received sham CPAP. To monitor adherence, a Respironics^®^ Encore^®^ Pro SmartCard^®^ was used, and adherence data from the two months leading up to follow-up visits were collected and analyzed.

### Participants

The criteria for participant inclusion and exclusion have been previously outlined (Kushida et al., 2012). Inclusion criteria included: (1) an apnea hypopnea index (AHI) ≥ 10 as determined by polysomnography (PSG); (2) age ≥ 18 years. The main exclusion criteria were: (1) prior use of CPAP, oxygen desaturation on PSG below 75% for more than 10% of the recording time; (2) history of a motor vehicle accident attributed to sleepiness, presence of several chronic medical conditions, and use of medications potentially impacting sleep or neurocognitive function.

### Scoring of respiratory events

Apneas and hypopneas were scored based on the American Academy of Sleep Medicine (1999) criteria. An apnea was characterized by a reduction of more than 90% in nasal pressure signal amplitude from baseline, lasting at least 10 s. Hypopneas were defined as either a reduction in nasal pressure signal amplitude between 50 and 90% of baseline or a less pronounced reduction that did not meet this threshold but was accompanied by an oxygen desaturation greater than 3% or an arousal, with a minimum duration of 10 s. The AHI was determined by calculating the total number of apneic and hypopneic events per hour of sleep.

### Cardiopulmonary coupling analysis

The approach for analyzing APPLES data using cardiopulmonary coupling (CPC) has been described in previous publications (Thomas et al., 2007; Thomas et al., 2005; Al Ashry et al., 2021). Briefly, heart rate variability (HRV) and electrocardiogram (ECG)—derived respiration were extracted from a single-channel ECG recorded during the overnight polysomnogram. ECG-derived respiration reflects amplitude variations in the QRS complex caused by shifts in the cardiac electrical axis during breathing and thoracic impedance changes due to lung expansion and contraction. CPC analysis identifies three distinct patterns: high-frequency coupling (HFC; 0.1–0.4 Hz), indicative of stable breathing during NREM sleep; low-frequency coupling (LFC; 0.01 to < 0.1 Hz), associated with apneas and hypopneas; and very low-frequency coupling (v-LFC; 0 to < 0.01 Hz), observed during wakefulness and REM sleep. Within the LFC domain, two subtypes are recognized. The first, e-LFC_NB_, is characterized by a narrow-spectral-band dispersion of CPC spectral peaks between 0.006 and 0.1 Hz, suggesting the presence of central sleep apnea, periodic breathing, or complex sleep apnea. The second, e-LFC_BB_, exhibits a broad-spectral-band pattern and is linked to OSA. These CPC metrics are quantified as the percentage of analysis windows relative to the total sleep period.

### Neurocognitive function

This study primarily utilizes three key neurocognitive variables, each representing a distinct neurocognitive domain: (1) Pathfinder Number Test-Total Time (PFN-TOTL): participants connect numbers in sequence on a computer, with a shorter total time indicating better attention and psychomotor function; (2) Buschke Selective Reminding Test-Sum Recall (BSRT-SR): participants recall words across 6 trials, with a higher total number of recalled words indicating better verbal learning and memory function, and (3) Sustained Working Memory Test-Overall Mid-Day Index (SWMT-OMD): participants compare the spatial position of stimuli and press corresponding buttons, with the overall index combining behavioral performance and EEG data to assess executive and frontal-lobe function, where a higher index indicates better performance. The detailed measurement procedures for each variable have been outlined in prior studies (Kushida et al., 2012). All three variables were evaluated during the baseline and at the 2-month and 6-month follow-up visits.

### Statistical analysis

Data summaries are presented as mean±standard deviation [SD] for normally distributed data, or as median [IQR] for non-normally distributed data. ROC curves were constructed for the improvements in SWMT-OMD, BSRT-SR, and PFN-TOTL at the 2 month and 6 month post-treatment follow-ups. These optimal cutoffs were determined using Youden’s J index. The selection of either a two-sample t-test or Wilcoxon rank-sum test for comparing measurements between the two groups was based on the data’s distribution normality. Categorical comparisons were conducted using Chi-Square and Fisher exact tests. To identify predictors of neurocognitive function changes, a backward elimination logistic regression analysis was applied, adjusted for study site, CPAP adherence, age, gender, body mass index (BMI), and OSA severity. Statistical analyses were performed using SPSS 19 and Origin Pro 8. All tests were two-tailed, and a *p*-value of less than 0.05 was considered statistically significant.

## Result

### Study population

A total of 1,516 participants were enrolled in the APPLES, starting in November 2003 and studied for up to 6-months over 11 visits, of which 1,104 were randomized to active vs. sham CPAP (REMstar Pro, Philips Respironics, Inc.) devices; 555 subjects were allocated to CPAP group, and 362 had successful CPC analysis, for the 549 subjects with sham CPAP, 337 had CPC results. Studies were excluded from the CPC analysis as a result of having ECG signal issues (e.g., arrhythmias, excessive noise, poor signal quality, data dropouts), discrepancies between the data-file recording time and that reported on the PSG, and/or unsuccessful processing. In the study, we successfully collected baseline and 2-month follow-up SWMT-OMD data from 230 participants, while 306 participants provided complete data spanning from baseline to 6-months (see Supplementary Table S1). Similarly, for the BSRT-SR, we obtained baseline and 2-month data from 239 participants (see Supplementary Table S2), and baseline to 6-month data from 314 participants. By applying the Youden index analysis, we identified several critical cutoff values for e-LFC_NB_%: For predicting an increase in SWMT-OMD, an e-LFC_NB_% threshold of > 2.35% optimally corresponds to a > 0.6 increase after 2 months, while a threshold of > 9.45% is optimal for a > 0.8 increase after 6 months. Similarly, for BSRT-SR, an e-LFC_NB_% of > 3.65% serves as the optimal cutoff for a 12-point increase after 2 months, whereas a threshold of > 2.15% corresponds to a 4-point increase after 6 months.

### Baseline characteristics

In the cohort of subjects analyzed, the median age was 52.46 ± 12.43 years, and their BMI was 32.69 ± 7.75 kg/m^2^. Regarding the SWMT-OMD assessments, out of 230 subjects who provided data at 2-months, 86 individuals exhibited an e-LFC_NB_% > 2.35%; whereas among the 306 subjects with data available at 6-months, 50 individuals showed an e-LFC_NB_% > 9.45%. For the BSRT-SR evaluations, of the 239 subjects with 2-month data, 64 had an e-LFC_NB_% > 3.65%; and among the 314 subjects with 6-month data, 133 demonstrated an e-LFC_NB_% > 2.15%.

### Neurocognitive function responses

**Figure 1:**
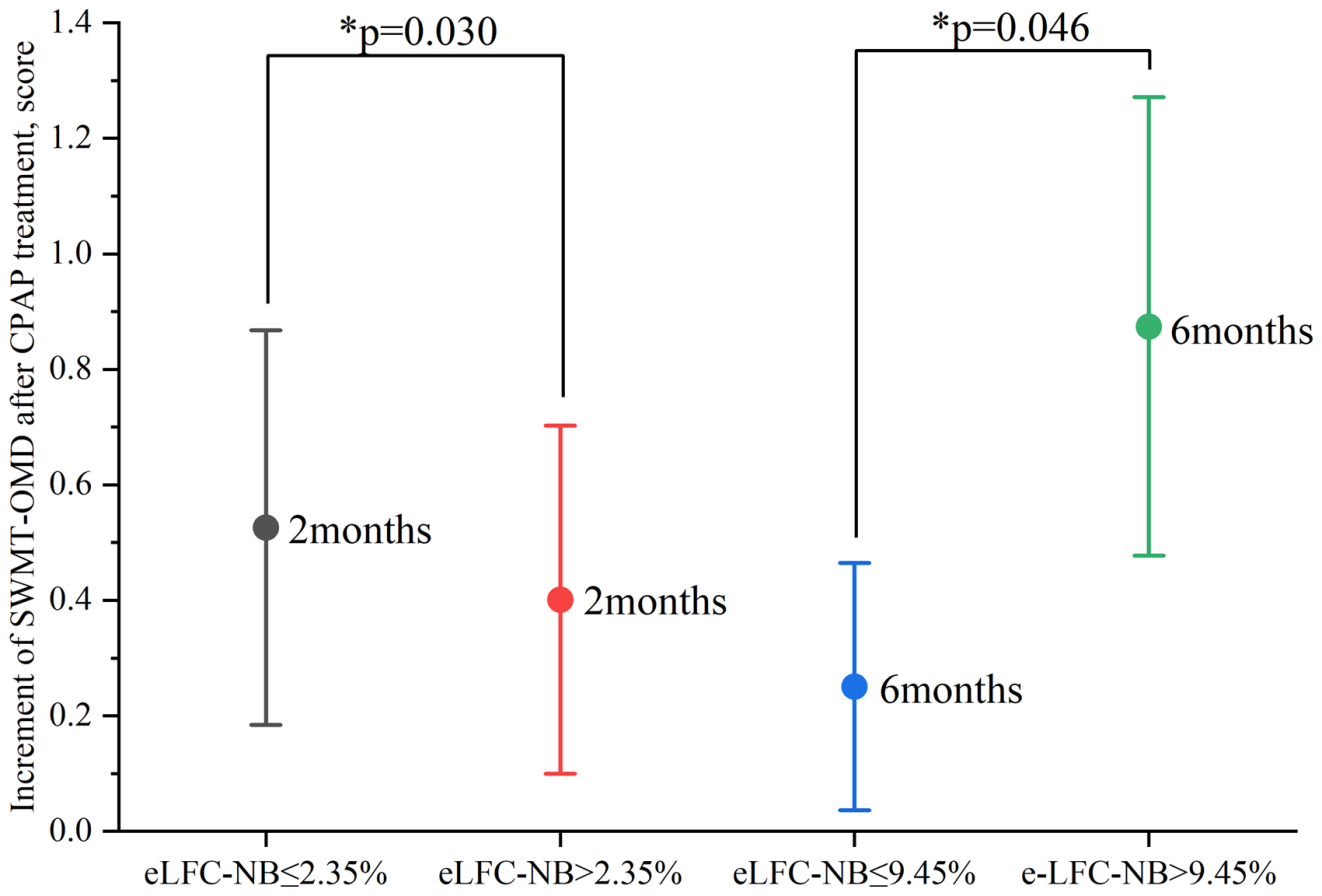
Improvement in SWMT-OMD after 2 and 6 months of CPAP treatment. CPAP: continuous positive airway pressure; SWMT-OMD: Sustained Working Memory Test-Overall Mid-Day Index. Improvement in SWMT-OMD as indicated by the test score after CPAP treatment minus the baseline test score. The circle represents the mean value. Data are presented as mean ± 95% confidence interval. *Statistically significant greater improvement in the high e-LFC_NB_ groups.

**Figure 2:**
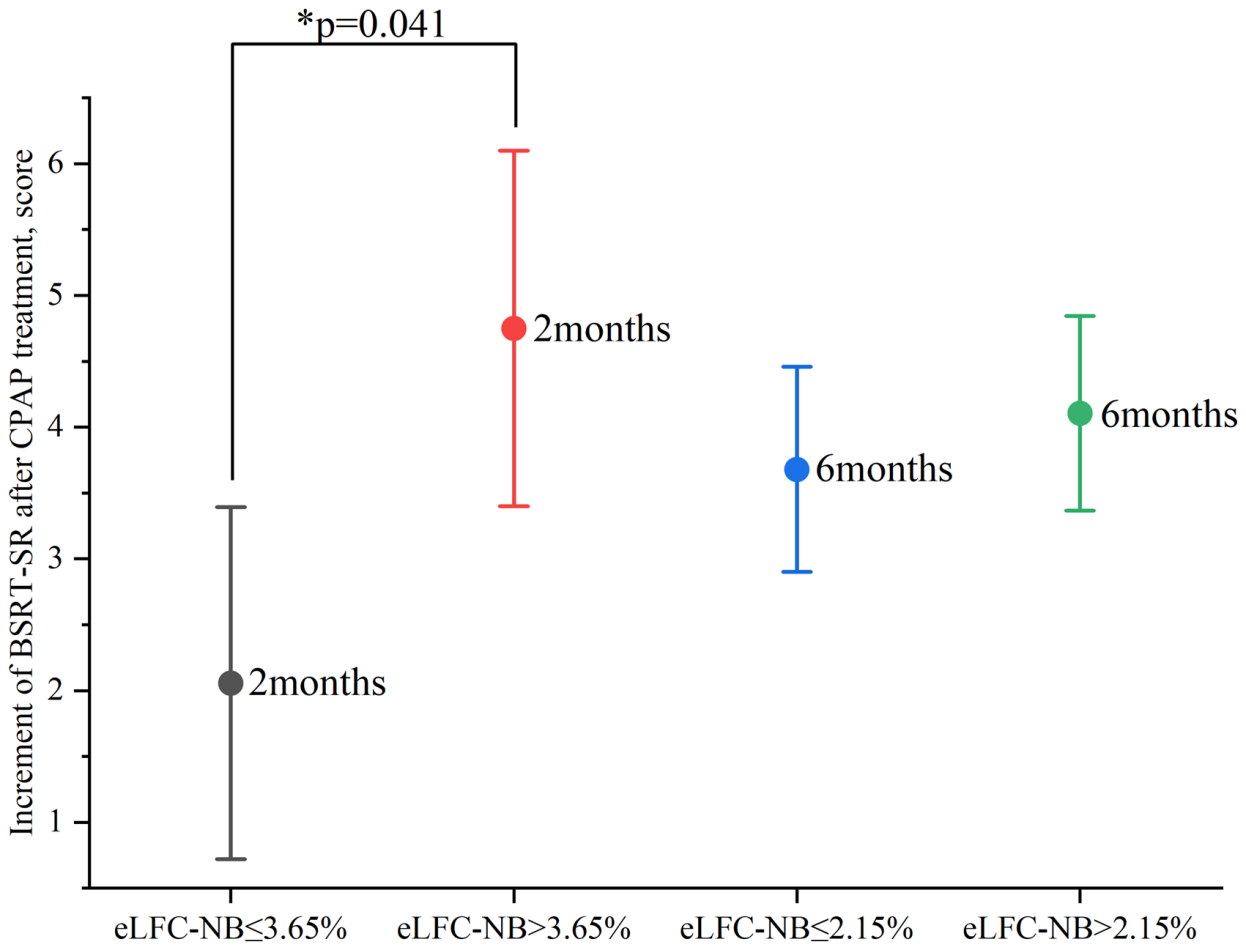
Improvement in BSRT-SR after 2 and 6 months of CPAP treatment. CPAP: continuous positive airway pressure; BSRT-SR: Buschke Selective Reminding Test-Sum Recall. Improvement in SWMT-OMD as indicated by the test score after CPAP treatment minus the baseline test score. The circle represents the mean value. Data are presented as mean ± 95% confidence interval. *Statistically significant greater improvement in the high e-LFC_NB_ groups after 2 months of treatment.

After two months of treatment, a greater proportion of participants in the higher e-LFC_NB_% groups showed significant cognitive improvement. Specifically, among the 86 subjects with e-LFC_NB_% > 2.35%, 21 participants (24.4% vs. 13.2%) exhibited an increase of more than 0.65 in SWMT-OMD test scores (see Table 1). Similarly, among the 64 subjects with e-LFC_NB_% > 3.65%, 21 participants (32.8% vs. 17.1%) demonstrated an improvement of more than 12 points in BSRT-SR test scores (see Table 2). After six months of treatment, the trend remained: among the 50 subjects with e-LFC_NB_% > 9.45% group, 12 participants (24% vs. 10.9%) experienced an increase of more than 0.8 in SWMT-OMD test scores (see Table 1). Figures 1, 2 show the extent of improvement in SWMT-OMD and BSRT-SR after 2 and 6 months of CPAP treatment. In contrast, among participants receiving sham CPAP, no significant differences in neurocognitive function improvements were observed across different e-LFC_NB_% groups at either the 2-month or 6-month assessments (see Supplementary Tables S3, S4).

**Table 1:** Characteristics of subjects divided by different e-LFC_NB_ % on CPAP (SWMT-OMD groups).

	Overall (*n* = 362)	e-LFC_NB_ % ≤ 2.35% (*n* = 144)	e-LFC_NB_ % > 2.35% (*n* = 86)	*p*	e-LFC_NB_ % ≤ 9.45% (*n* = 256)	e-LFC_NB_ % > 9.45% (*n* = 50)	*p*
Age (years)	52.46 ± 12.43	51.18 ± 13.05	55.02 ± 12.38	0.029	51.83 ± 12.27	54.84 ± 11.96	0.112
Male (%)	247 (68.2%)	89 (61.8%)	67 (77.9%)	0.011	161 (62.9%)	47 (94%)	< 0.001
Race				0.518			0.085
Native American (%)	2 (0.6%)	-	-		1 (0.4%)	1 (2%)	
Asian (%)	24 (6.6%)	9 (6.3%)	8 (9.3%)		12 (4.7%)	7 (14%)	
Black (%)	23 (6.4%)	6 (4.2%)	7 (8.1%)		15 (5.9%)	4 (8%)	
Hispanic (%)	24 (6.6%)	10 (6.9%)	4 (4.7%)		19 (7.4%)	2 (4%)	
White (%)	288 (79.6%)	118 (81.9%)	67 (77.9%)		208 (81.3%)	36 (72%)	
Other (%)	1 (0.3%)	1 (0.7%)	-		1(0.4%)	-	
BMI Kg/m^2^	32.69 ± 7.75	31.77 ± 7.00	33.85 ± 8.31	0.085	32.61 ± 7.87	33.65 ± 7.85	0.398
AHI /hour	33.25 [18.38–55.03]	24.30 [16.20–39.18]	51.35 [32.38–75.60]	< 0.001	29.05 [17.18–49.08]	66.75 [52.58–82.91]	< 0.001
AHI<15 (%)	57 (15.7%)	31 (21.5%)	5 (5.8%)	0.002	48 (18.8%)	-	0.001
AHI ≥ 15 (%)	305 (84.3%)	113 (78.5%)	81 (94.2%)		208 (81.3%)	50 (100%)	
Alcohol (%)	245 (67.7%)	103 (71.5%)	56 (65.1%)	0.308	175 (68.4%)	34 (68%)	0.960
Current smoker (%)	44 (12.2%)	18 (12.5%)	10 (11.6%)	0.830	33 (12.9%)	4 (8%)	0.332
SWMT-OMD score	−0.44 ± 2.82	−0.54 ± 3.01	−0.35 ± 2.60	0.466	−0.17 ± 1.80	−0.64 ± 3.39	0.603
Change after 2 months	0.03 [−0.42–0.45]	0 [−0.38–0.44]	0.08 [−0.63–0.63]	0.815	-	-	-
Change after 2 months > 0.65 (%)	40 (17.4%)	19 (13.2%)	21 (24.4%)	0.030	-	-	-
Change after 6 months	0.08 [−0.35–0.53]	-	-	-	0.08 [−0.35–0.51]	0.08 [−0.34–0.81]	0.398
Change after 6 months > 0.8 (%)	40 (13.1%)	-	-	-	28 (10.9%)	12 (24%)	0.012
BSRT-SR score	49.97 ± 8.95	-	-	-	-	-	-
Change after 2 months	3 [−6–11]	-	-	-	-	-	-
Change after 2 months > 12 (%)	51 (21.3%)	-	-	-	-	-	-
Change after 6 months	3.5 [−1–8]	-	-	-	-	-	-
Change after 6 months > 4 (%)	139 (44.3%)	-	-	-	-	-	-
PFN-TOTL, score	24.28 ± 6.56	-	-	-	-	-	-
Change after 2 months	0.52 [−4.9–6.47]	-	-	-	-	-	-
Change after 6 months	−0.15 [−2.19–2.11]	-	-	-	-	-	-

AHI: apnea hypopnea index; BMI: body mass index; BSRT-SR: Buschke Selective Reminding Test-Sum Recall; e-LFC_NB_: elevated low frequency coupling, narrow-band; PFN-TOTL: Pathfinder Number Test-Total Time; SWMT-OMD: Sustained Working Memory Test-Overall Mid-Day Index.

**Table 2:** Characteristics of subjects divided by different e-LFC_NB_ % on CPAP (BSRT-SR groups).

	Overall (*n* = 362)	e-LFC_NB_ % ≤ 3.65% (*n* = 175)	e-LFC_NB_ % > 3.65% (*n* = 64)	*p*	e-LFC_NB_ % ≤ 2.15% (*n* = 181)	e-LFC_NB_ % > 2.15% (*n* = 133)	*p*
Age (years)	52.46 ± 12.43	51.62 ± 12.59	55.45 ± 13	0.040	50.77 ± 12.4	54.93 ± 11.67	0.003
Male (%)	247 (68.2%)	109 (62.3%)	51 (79.7%)	0.011	116 (64.1%)	100 (75.2%)	0.036
Race				0.958			0.060
Native American (%)	2 (0.6%)	-	-		1 (0.6%)	1 (0.8%)	
Asian (%)	24 (6.6%)	12 (6.9%)	5 (7.8%)		9 (5%)	11 (8.3%)	
Black (%)	23 (6.4%)	10 (5.7%)	4 (6.3%)		7 (3.9%)	12 (9%)	
Hispanic (%)	24 (6.6%)	12 (6.9%)	3 (4.7%)		16 (8.8%)	5 (3.8%)	
White (%)	288 (79.6%)	140 (80%)	52 (81.3%)		148 (81.8%)	103 (77.4%)	
Other (%)	1 (0.3%)	1 (0.6%)	-		-	1 (0.8%)	
BMI, Kg/m^2^	32.69 ± 7.75	31.79 ± 6.94	34.58 ± 8.46	0.027	32.19 ± 8.04	33.59 ± 7.49	0.061
AHI /hour	33.25 [18.38–55.03]	25.5 [16.5–42.8]	54.75 [39.82–78.05]	< 0.001	23.2 [15.35–41.8]	52.7 [32.9–73.35]	< 0.001
AHI<15 (%)	57 (15.7%)	36 (20.6%)	139 (79.4%)	< 0.001	44 (24.3%)	5 (3.8%)	< 0.001
AHI ≥ 15 (%)	305 (84.3%)	-	64 (100%)		137 (75.7%)	128 (96.2%)	
Alcohol (%)	245 (67.7%)	125 (71.4%)	39 (60.9%)	0.122	131 (72.4%)	84 (63.2%)	0.082
Current smoker (%)	44 (12.2%)	23 (13.1%)	7 (10.9%)	0.649	21 (11.6%)	17 (12.8%)	0.751
SWMT-OMD score	−0.44 ± 2.82	-	-	-	-	-	-
Change after 2 months	0.03 [−0.42–0.45]	-	-	-	-	-	-
Change after 2 months > 0.65(%)	40 (17.4%)	-	-	-	-	-	-
Change after 6 months	0.08 [−0.35–0.53]	-	-	-	-	-	-
Change after 6 months > 0.8(%)	40 (13.1%)	-	-	-	-	-	-
BSRT-SR score	49.97 ± 8.95	50.56 ± 8.56	48.56 ± 9.61	0.142	51.01 ± 8.56	48.86 ± 8.53	0.028
Change after 2 months	3 [−6–11]	3 [−7–10]	5 [−3.75–14]	0.128	-	-	-
Change after 2 months > 12(%)	51 (21.3%)	30 (17.1%)	21 (32.8%)	0.009	-	-	-
Change after 6 months	3.5 [−1–8]	-	-	-	3 [−1–8]	4 [−1–8]	0.491
Change after 6 months > 4(%)	139 (44.3%)	-	-	-	73 (40.3%)	66 (49.6%)	
PFN-TOTL, score	24.28 ± 6.56	-	-	-	-	-	-
Change after 2 months	0.52 [−4.9–6.47]	-	-	-	-	-	-
Change after 6 months	−0.15 [−2.19–2.11]	-	-	-	-	-	-

AHI: apnea hypopnea index; BMI: body mass index; BSRT-SR: Buschke Selective Reminding Test-Sum Recall; e-LFC_NB_: elevated low frequency coupling, narrow-band; PFN-TOTL: Pathfinder Number Test-Total Time; SWMT-OMD: Sustained Working Memory Test-Overall Mid-Day Index.

### Logistic regression

In the in-depth exploration of logistic regression analysis, we unveiled a significant positive correlation between e-LFC_NB_% levels and the improvement in neurocognitive function following CPAP treatment. Specifically, when e-LFC_NB_% > 2.35%, there is a significant increase in the probability of SWMT-OMD test score improvement > 0.65 after 2-months CPAP treatment (OR: 2.617, 95% CI: 1.095–6.252, *p*: 0.030) (see Table 3); similarly, e-LFC_NB_% > 3.65% is associated with an improvement > 12 in BSRT-SR test score (OR: 2.696, 95% CI: 1.041–6.982, *p*: 0.041) (see Table 4). Furthermore, after 6 months CPAP treatment, individuals with e-LFC_NB_% > than 9.45% is related with higher rate of SWMT-OMD score improvement > 0.8 (OR: 2.553, 95% CI: 1.017–6.409, *p*: 0.046) (see Table 3). However, for the improvement in BSRT-SR after 6 months CPAP treatment (*p*: 0.541) and the overall improvement in PFN-TOTL (2-month *p*, 0.655 6-month *p*, 0.548), we found no evidence of association with e-LFC_NB_% (see Supplementary Tables S4, S5). Additionally, in predicting the response of sham CPAP on neurocognitive function, e-LFC_NB_% did not demonstrate any effect (see Supplementary Tables S6–S8).

**Table 3:** Logistic regression model for SWMT-OMD increase after 2 months and 6 months treatment.

2 months after CPAP	SWMT-OMD change > 0.65		
	OR	95% CI	*p*
Model 1: e-LFC_NB_ > 2.35%	2.216	1.067–4.234	0.032
Model 1 + study site + baseline SWMT-OMD	2.865	1.295–6.340	0.009
Model 2 + adherence	2.617	1.095–6.252	0.030
Model 3 + age, sex, BMI	2.617	1.095–6.252	0.030
Model 4 + AHI	2.617	1.095–6.252	0.030

6 months after CPAP	SWMT-OMD change > 0.8		
	OR	95% CI	*p*
Model 1: e-LFC_NB_ > 9.45%	2.571	1.204–5.490	0.015
Model 1 + study site + baseline SWMT-OMD	2.456	1.019–5.919	0.045
Model 2 + adherence	2.553	1.017–6.409	0.046
Model 3 + age, sex, BMI	2.553	1.017–6.409	0.046
Model 4 + AHI	2.553	1.017–6.409	0.046

AHI: apnea hypopnea index; BMI: body mass index; e-LFC_NB_: elevated low frequency coupling, narrow-band; SWMT-OMD: Sustained Working Memory Test-Overall Mid-Day Index.

**Table 4:** Logistic regression model for BSRT-SR increase after 2 months and 6 months treatment.

2 months after CPAP	BSRT-SR change > 12		
	OR	95% CI	*p*
Model 1: e-LFC_NB_ > 3.65%	2.360	1.228–4.537	0.010
Model 1 + study site + baseline BSRT-SR	2.206	0.901–5.402	0.083
Model 2 + adherence	2.696	1.041–6.982	0.041
Model 3 + age, sex, BMI	2.696	1.041–6.982	0.041
Model 4 + AHI	2.696	1.041–6.982	0.041

6 months after CPAP	BSRT-SR change > 4		
	OR	95% CI	*p*
Model 1: e-LFC_NB_ > 2.15%	-	-	0.101
Model 1 + study site + baseline BSRT-SR	-	-	0.359
Model 2 + adherence	-	-	0.970
Model 3 + age, sex, BMI	-	-	0.541
Model 4 + AHI	-	-	0.541

AHI: apnea hypopnea index; BMI: body mass index; BSRT-SR: Buschke Selective Reminding Test-Sum Recall; e-LFC_NB_: elevated low frequency coupling, narrow-band.

## Discussion

This study uncovered the following findings: (1) a positive correlation between e-LFC_NB_% and short-term improvements in BSRT-SR among OSA patients after CPAP treatment; (2) a positive correlation between e-LFC_NB_% and both short-term and long-term improvements in SWMT-OMD among OSA patients following CPAP treatment. These findings may help predict which individuals are likely to experience enhancements in neurocognitive function from OSA therapy, as indicated by the sequence: apnea with e-LFC_NB_%/High Loop Gain → NREM instability/fragmentation + Sympathetic Activation + Hypoxia → Prefrontal/Hippocampal Dysfunction → Specific Cognitive Deficits → Reversal by CPAP.

The pathogenic mechanisms through which OSA impairs neurocognitive functions include: intermittent hypoxemia, cytokine activation, metabolic dysfunction, sleep deprivation, hypercapnia and sympathetic nervous system activation (Pollicina et al., 2021). As high loop gain sleep apnea is NREM-dominant, the predominance of respiratory events in NREM sleep may cause disproportionate sleep fragmentation, which is crucial in the development of neurocognitive function (Devita et al., 2019). NREM sleep enhances information encoding capacity and learning in neural networks through a complex interplay of slow oscillations, spindles, and sharp-wave ripples (Mizuseki and Miyawaki, 2017; Peyrache and Seibt, 2020; Salfi et al., 2020), and is at risk from sleep fragmentation. Even brief periods of NREM sleep can significantly boost and restore cognitive functions (Kharas et al., 2024) but substantial fragmentation is likely to adversely impact memoy and learning functions. Sleep fragmentation itself can impair metabolic function (Stamatakis and Punjabi, 2010). Chronic sleep fragmentation exposures in a murine model mimicking the fragmentation of sleep that characterizes patients with apnea elicits evidence of inflammation in brain regions and explicit memory impairments in mice (Puech et al., 2023). In this model, sleep fragmentation was also associated with increased BBB permeability, the magnitude of which was closely associated with cognitive functional loss (Puech et al., 2023). Apneas and intermittent hypoxia events experienced during NREM sleep can trigger the activation of the sympathetic nervous system, leading to endothelial dysfunction (Lambert et al., 2013; Swenson, 2020) and blood pressure surges (Narkiewicz et al., 1998; Peled et al., 1998). Moreover, hypoxic events themselves can cause direct damage to neurocognitive functions (He et al., 2024; Chokesuwattanaskul et al., 2021).

Previous studies have confirmed that e-LFC_NB_% is associated with the severity of OSA, sleep stability, respiratory chemoreflex regulation, and intermittent hypoxia (Deacon and Catcheside, 2015; Ibrahim et al., 2010; Thomas et al., 2018). Hypoxic events stimulate the sympathetic nervous system, thus e-LFC_NB_% is also a marker of carotid body activation (Joyner et al., 2018). However, we adjusted for apnea severity in our analysis. CPAP treatment can improve neurocognitive function by reversing the processes of chronic intermittent hypoxia and sympathetic nerve activation, as well as reducing sleep fragmentation and can even slightly increase the duration of sleep, improving neural structural adaptability, and facilitating neuronal repair (Ziegler et al., 2001; Sánchez et al., 2009). It was recently reported using the APPLES that a higher arousal threshold is associated with greater improvements in executive function following CPAP therapy (Zinchuk et al., 2025). That is a lower propensity for fragmentation was protective.

In this study, two primary outcome indicators reflecting neurocognitive functions were assessed. First, SWMT-OMD primarily evaluates individuals’ executive functions and frontal lobe functions, particularly abilities related to working memory, and is significantly associated with the prefrontal cortex. Second, BSRT-SR mainly assesses individuals’ learning and memory capabilities, specifically verbal learning and memory, and is related to the brain’s hippocampus and temporal cortex (Kushida et al., 2012; Pollicina et al., 2021). Studies have demonstrated that the SWMT is sensitive to subtle changes in executive function (Gevins et al., 2011), and improvements in SWMT-OMD reflect enhanced frontal lobe performance. Similarly, research has indicated that improvements in BSRT-SR scores (even by 4–6 points) are clinically significant in OSA populations (Beebe et al., 2003). In our study, the observed improvements (e.g., BSRT-SR > 12) substantially exceed these benchmarks, underscoring their functional relevance. While the hippocampus is more sensitive to hypoxia (Churilova et al., 2022), its ability to adapt to prolonged hypoxic exposure is greater than that of the prefrontal cortex, potentially providing compensatory mechanisms (Shaw et al., 2021). During NREM hypoxia, sympathetic activation leads to increased levels of norepinephrine, which impairs the prefrontal cortex’s ability to support higher-order cognitive functions, such as decision-making and working memory (Arnsten, 2009). This makes the prefrontal cortex particularly susceptible to impairment from sympathetic activation during sleep. As a biomarker of sympathetic nerve excitation and NREM-period hypoxia, e-LFC_NB_% could plausibly predict improvements in neurocognitive function after treatment. For BSRT-SR, the relatively slow subsequent improvement of the hippocampus and its weaker correlation with sympathetic excitation and hypoxia compared to the prefrontal cortex may explain why it can only predict short-term effects. In addition, the inconsistency in the quality of the BSRT-SR assessment scales before and after treatment, as mentioned in previous studies (Kushida et al., 2012), may also account for the results of this study. Regarding the third neurocognitive function assessment indicator mentioned in this study: PFN-TOTL, which is primarily used to evaluate a patient’s attention and psychomotor functions, is associated with the function of the prefrontal cortex (Braver et al., 2009). However, the study results indicate that e-LFC_NB_% does not effectively predict the degree of improvement in this indicator. This may be due to the sensitivity of the indicator to CPAP treatment being insufficient, rendering it a less effective marker for assessing neurocognitive function in patients with OSA (Kushida et al., 2012).

This analysis has several limitations that should be taken into account. Data loss occurred due to issues with signal quality and analysis. We were unable to assess the night-to-night stability of e-LFC_NB_%. Maintaining consistent quality in the assessment scales for neurocognitive function indicators could provide clearer indications of treatment responses. We did not directly estimate the loop gain in the analyzed data. Additionally, the subjective nature of cognitive function assessments may have influenced the results, as factors such as age, education, and social function status can affect individuals’ self-reports of cognitive function. The complex relationship between cognitive function and brain structure/function, including the brain’s compensatory and repair mechanisms, adds to the uncertainty in accurately evaluating cognitive impairment. Future research should address these limitations by employing larger, more diverse samples, and longitudinal study designs to better understand the impact of LFC_NB_% and other biomarkers on the improvement of neurocognitive function in OSA patients after CPAP treatment.

## Conclusion

Assessing pathological activation of the respiratory chemoreflex may help identify a biomarker for predicting improvement in neurocognitive function following CPAP treatment.

## Data Availability

The original contributions presented in the study are included in the article/Supplementary material, further inquiries can be directed to the corresponding author.

## Glossary

AHI: Apnea Hypopnea Index
APPLES: Apnea Positive Pressure Long-term Efficacy Study
BMI: Body Mass Index
BSRT-SR: Buschke Selective Reminding Test-Sum Recall
CI: Confidence Interval
CPAP: Continuous Positive Airway Pressure
CPC: Cardiopulmonary Coupling
e-LFC_NB_: Percentage of Narrow Band During Low Frequency Coupling
ECG: electrocardiogram
HFC: High frequency coupling
HRV: heart rate variability
IQR: interquartile range
LFC: Low Frequency Coupling
NREM: Non Rapid Eye Movement Sleep
OSA: Obstructive Sleep Apnea
PFN-TOTL: Pathfinder Number Test-Total Time
PSG: Polysomnogram
REM: Rapid Eye Movement Sleep
SD: Standard Deviation
SWMT-OMD: Sustained Working Memory Test-Overall Mid-Day Index
