# Correction: Pathological respiratory chemoreflex activation predicts improvement of neurocognitive function in response to continuous positive airway pressure therapy

**DOI:** 10.3389/fnins.2025.1714129

**Published:** 2025-10-13

**Authors:** Yu-Tong Hu, Yue-Nan Ni, Hugi Hilmisson, Robert Joseph Thomas

**Affiliations:** ^1^Department of Respiratory, Critical Care and Sleep Medicine, West China School of Medicine and West China Hospital, Sichuan University, Chengdu, China; ^2^Department of Respiratory Care, West China School of Medicine and West China Hospital, Sichuan University, Chengdu, China; ^3^MyCardio-LLC, Denver, CO, United States; ^4^Division of Pulmonary, Critical Care and Sleep Medicine, Beth Israel Deaconess Medical Center, Boston, MA, United States

**Keywords:** sleep apnea, positive airway pressure, cardiopulmonary coupling, neurocognitive function, high loop gain

The title of this article was erroneously given as: “Pathological respiratory chemoreflex activation predicts improvement of neurocognitive function in response to contimuous positive airway pressure therapy”. The correct title of the article is “Pathological respiratory chemoreflex activation predicts improvement of neurocognitive function in response to continuous positive airway pressure therapy.”

An extraneous period was present after the citation “(Lv et al., 2023).”

A correction has been made to the section Background, second paragraph:

“Continuous positive airway pressure (CPAP), which is regarded as the first line therapy for OSA, can improve hypoxia in OSA patients, reduce sleep fragmentation, and reverse sympathetic nerve excitation (Patil et al., 2019), but there is controversy regarding whether CPAP can improve cognitive dysfunction caused by OSA (Bucks et al., 2013). The existence of complex pathologies especially high loop gain maybe a reason for the failure due to impairment in both effectiveness and adherence (Ni and Thomas, 2023; Cheng et al., 2024). It is also plausible that pathological respiratory chemoreflex activation and the resulting heightened sympathetic activation and other pleotrophic effects such as oxidative stress, inflammatory response, and neuronal damage (Lv et al., 2023) may influence development of neurocognitive dysfunction, and response to treatment.”

The word “imfluences” was misspelled.

A correction has been made to the section Background, fourth paragraph:

“As described in our previous work, narrow band elevated low frequency coupling (e-LFCNB) measured by cardiopulmonary coupling sleep spectrograms is a biomarker indicating periodic breathing and central sleep apnea (Thomas et al., 2007), indicating high loop gain. Our previous study showed that e-LFCNB was a strong predictor for blood pressure dropping after CPAP in OSA (Ni et al., 2024). However, whether it influences neurocognitive function improvement by CPAP is not known.”

The original version of this article has been updated.

