# Supplementary material for: Pathological respiratory chemoreflex activation predicts improvement of neurocognitive function in response to continuous positive airway pressure therapy
Source: Front Neurosci. 2025 Sep 24;19:1619467. doi: 10.3389/fnins.2025.1619467 (PMC12504490; doi:10.3389/fnins.2025.1619467)
Supplement: Supplementary file 1 [file Table_1.docx]

| **Table 1s: Characteristics of subjects divided by different SWMT-OMD on CPAP** | | | | | | | |
| --- | --- | --- | --- | --- | --- | --- | --- |
|  | **Overall（n = 362）** | **SWMT-OMD change after 2 months ≤ 0.65 (n = 190)** | **SWMT-OMD change after 2 months > 0.65 (n = 40)** | **p** | **SWMT-OMD change after 6 months ≤ 0.8 (n = 266)** | **SWMT-OMD change after 6 months > 0.8 (n = 40)** | **p** |
| Age (years) | 52.46 ± 12.43 | 52.24 ± 13.38 | 54.40 ± 10.39 | 0.338 | 51.89 ± 12.42 | 55.15 ± 10.73 | 0.117 |
| Male (%) | 247 (68.2%) | 124 (65.3%) | 32 (80%) | 0.07 | 181 (68%) | 27 (67.5%) | 0.945 |
| Race |  |  |  | 0.115 |  |  | 0.420 |
| Native American (%) | 2 (0.6%) | - | - |  | 2 (0.8%) | - |  |
| Asian (%) | 24 (6.6%) | 15 (7.9%) | 2 (5%) |  | 16 (6%) | 3 (7.5%) |  |
| Black (%) | 23 (6.4%) | 10 (5.3%) | 3 (7.5%) |  | 14 (5.3%) | 5 (12.5%) |  |
| Hispanic (%) | 24 (6.6%) | 8 (4.2%) | 6 (15%) |  | 20 (7.5%) | 1 (2.5%) |  |
| White (%) | 288 (79.6%) | 156 (82.1%) | 29 (72.5%) |  | 213 (80.1%) | 31 (77.5%) |  |
| Other (%) | 1 (0.3%) | 1 (0.5%) | - |  | 1(0.4%) | - |  |
| BMI Kg/m^2^ | 32.69 ± 7.75 | 32.39 ± 7.74 | 33.30 ± 6.71 | 0.309 | 32.89 ± 7.85 | 32.06 ± 8.01 | 0.521 |
| AHI /hour | 33.25 [18.38-55.03] | 30.95 [18.28-50.98] | 41.30 [20.98-80.50] | 0.065 | 33.25 [18.28-54.15] | 46.90 [21.95-71.93] | 0.018 |
| AHI＜15 (%) | 57 (15.7%) | 31 (16.3%) | 5 (12.5%) | 0.546 | 46 (17.3%) | 2 (5%) | 0.046 |
| AHI ≥ 15 (%) | 305 (84.3%) | 159 (83.7%) | 35 (67.5%) |  | 220 (82.7%) | 38 (95%) |  |
| Alcohol (%) | 245 (67.7%) | 132 (69.5%) | 27 (67.5%) | 0.806 | 184 (69.2%) | 25 (62.5%) | 0.398 |
| Current smoker (%) | 44 (12.2%) | 25 (13.2%) | 3 (7.5%) | 0.466 | 33 (12.4%) | 4 (10%) | 0.861 |
| e-LFC_NB_ | 5.72 ± 12.7 | 4.68 ± 10.98 | 6.83 ± 11.91 | 0.106 | 5.25 ± 12.14 | 9.21 ± 16.37 | 0.113 |
| e-LFC_NB_ > 2.35% (%) | 132 (36.5%) | 65 (34.2%) | 21 (52.5%) | 0.030 | - | - | - |
| e-LFC_NB_ > 9.45% (%) | 59 (16.3%) | - | - | - | 38 (14.3%) | 12 (30%) | 0.012 |

AHI: apnea hypopnea index; BMI: body mass index; e-LFC_NB_: elevated low frequency coupling, narrow-band; SWMT-OMD: Sustained Working Memory Test-Overall Mid-Day Index

| **Table 2s: Characteristics of subjects divided by different BSRT-SR on CPAP** | | | | | |
| --- | --- | --- | --- | --- | --- |
|  | **Overall（n = 362）** | **BSRT-SR change after 2 months ≤ 12 (n = 188)** | **BSRT-SR change after 2 months > 12 (n = 51)** | **p** |  |
| Age (years) | 52.46 ± 12.43 | 51.53 ± 13.03 | 56.75 ± 11.03 | 0.012 |  |
| Male (%) | 247 (68.2%) | 121 (64.4%) | 39 (76.5%) | 0.103 |  |
| Race |  |  |  | 0.089 |  |
| Native American (%) | 2 (0.6%) | - | - |  |  |
| Asian (%) | 24 (6.6%) | 15 (8%) | 2 (3.9%) |  |  |
| Black (%) | 23 (6.4%) | 8 (4.3%) | 6 (11.8%) |  |  |
| Hispanic (%) | 24 (6.6%) | 13 (6.9%) | 2 (3.9%) |  |  |
| White (%) | 288 (79.6%) | 152 (80.9%) | 40 (78.4%) |  |  |
| Other (%) | 1 (0.3%) | - | 1(2%) |  |  |
| BMI Kg/m^2^ | 32.69 ± 7.75 | 32.53 ± 7.42 | 32.59 ± 7.70 | 0.905 |  |
| AHI /hour | 33.25 [18.38-55.03] | 33.20 [18.23-52.58] | 32.90 [22.40-61.70] | 0.526 |  |
| AHI＜15 (%) | 57 (15.7%) | 30 (16%) | 6 (11.8%) | 0.458 |  |
| AHI ≥ 15 (%) | 305 (84.3%) | 158 (84%) | 45 (88.2%) |  |  |
| Alcohol (%) | 245 (67.7%) | 134 (71.3%) | 30 (58.8%) | 0.089 |  |
| Current smoker (%) | 44 (12.2%) | 25 (13.3%) | 5 (9.8%) | 0.616 |  |
| e-LFC_NB_ | 5.72 ± 12.7 | 4.17 ± 9.68 | 7.76 ± 14.52 | 0.083 |  |
| e-LFC_NB_ > 3.65% (%) | 101 (27.9%) | 43 (22.9%) | 21 (41.2%) | 0.009 |  |

AHI: apnea hypopnea index; BMI: body mass index; BSRT-SR: Buschke Selective Reminding Test-Sum Recall; e-LFC_NB_: elevated low frequency coupling, narrow-band

| **Table 3s: Characteristics of subjects divided by different e-LFC_NB_ % in the control group** | | | | | | | |
| --- | --- | --- | --- | --- | --- | --- | --- |
|  | **Overall（n = 337）** | **e-LFC_NB_ % ≤ 2.35% (n = 108)** | **e-LFC_NB_ % > 2.35% (n = 86)** | **p** | **e-LFC_NB_ % ≤ 9.45% (n = 208)** | **e-LFC_NB_ % > 9.45% (n = 51)** | **p** |
| SWMT-OMD score | -0.57 ± 3 | -0.63 ± 3.24 | -0.06 ± 0.27 | 0.502 | -0.18 ± 1.63 | -0.03 ± 0.26 | 0.875 |
| Change after 2 months | 0.04 [-0.42-0.43] | -0.06 [-0.54-0.35] | 0.2 [-0.21-0.46] | 0.037 | - | - | - |
| Change after 2 months > 0.65 (%) | 31 (16%) | 18 (16.7%) | 13 (15.1%) | 0.770 | - | - | - |
| Change after 6 months | 0.08 [-0.38-0.56] | - | - | - | 0.10 [-0.36-0.58] | -0.01 [-0.51-0.40] | 0.281 |
| Change after 6 months > 0.8 (%) | 37 (14.3%) | - | - | - | 28 (13.5%) | 9 (17.6%) | 0.444 |
| BSRT-SR score | 49.92 ± 9.27 | - | - | - | - | - | - |
| Change after 2 months | 2 [-6-10.25] | - | - | - | - | - | - |
| Change after 2 months > 12(%) | 41 (19.9%) | - | - | - | - | - | - |
| Change after 6 months | 4 [0-8] | - | - | - | - | - | - |
| Change after 6 months > 4(%) | 131 (47.3%) | - | - | - | - | - | - |
| PFN-TOTL score | 23.98 ± 6.08 | - | - | - | - | - | - |
| Change after 2 months | 0.41 [-4.96-5.74] | - | - | -  - | - | - | -  - |
| Change after 6 months | -0.39 [-2.47-2.02] | - | - |  | - | - |  |

BSRT-SR: Buschke Selective Reminding Test-Sum Recall; e-LFC_NB_: elevated low frequency coupling, narrow-band; PFN-TOTL: Pathfinder Number Test-Total Time; SWMT-OMD: Sustained Working Memory Test-Overall Mid-Day Index

| **Table 4s: Characteristics of subjects divided by different e-LFC_NB_ % in the control group** | | | | | | | |
| --- | --- | --- | --- | --- | --- | --- | --- |
|  | **Overall（n = 337）** | **e-LFC_NB_ % ≤ 3.65% (n = 139)** | **e-LFC_NB_ % > 3.65% (n = 67)** | **p** | **e-LFC_NB_ % ≤ 2.15% (n = 162)** | **e-LFC_NB_ % > 2.15% (n = 115)** | **p** |
| SWMT-OMD score | -0.57 ± 3 | - | - | - | - | - | - |
| Change after 2 months | 0.04 [-0.42-0.43] | - | - | - | - | - | - |
| Change after 2 months > 0.65 (%) | 31 (16%) | - | - | - | - | - | - |
| Change after 6 months | 0.08 [-0.38-0.56] | - | - | - | - | - | - |
| Change after 6 months > 0.8 (%) | 37 (14.3%) | - | - | - | - | - | - |
| BSRT-SR score | 49.92 ± 9.27 | 50.60 ± 9.38 | 49.57 ± 8.28 | 0.310 | 50.17 ± 10.15 | 49.63 ± 8.16 | 0.347 |
| Change after 2 months | 2 [-6-10.25] | 1 [-7-9] | 4 [-4-13] | 0.105 | - | - | - |
| Change after 2 months > 12 (%) | 41 (19.9%) | 23 (16.5%) | 18 (26.9%) | 0.082 | - | - | - |
| Change after 6 months | 4 [0-8] | - | - | - | 4 [-0.25-8] | 4 [0-9] | 0.571 |
| Change after 6 months > 4 (%) | 131 (47.3%) | - | - | - | 76 (46.9%) | 55 (47.8%) | 0.881 |
| PFN-TOTL score | 23.98 ± 6.08 | - | - | - | - | - | - |
| Change after 2 months | 0.41 [-4.96-5.74] | - | - | - | - | - | - |
| Change after 6 months | -0.39 [-2.47-2.02] | - | - |  | - | - |  |

BSRT-SR: Buschke Selective Reminding Test-Sum Recall; e-LFC_NB_: elevated low frequency coupling, narrow-band; PFN-TOTL: Pathfinder Number Test-Total Time; SWMT-OMD: Sustained Working Memory Test-Overall Mid-Day Index

| **Table 5s: Logistic regression model for PFN-TOTL increase after 2 months and 6 months treatment** | | | |
| --- | --- | --- | --- |
| **2 months after CPAP** | **PFN-TOTL change > -5.8** | | |
|  | OR | 95% CI | p |
| **Model 1: e-LFC_NB_ > 2.35%** | 1.955 | 1.009-3.786 | 0.047 |
| **Model 1 + study site + baseline PFN-TOTL** | - | - | 0.950 |
| **Model 2 + adherence** | - | - | 0.761 |
| **Model 3 + age，sex，BMI** | - | - | 0.655 |
| **Model 4 + AHI** | - | - | 0.655 |
| **6 months after CPAP** | **PFN-TOTL change > -2.3** | | |
|  | OR | 95% CI | p |
| **Model 1: e-LFC_NB_ > 2.45%** | - | - | 0.133 |
| **Model 1 + study site + baseline PFN-TOTL** | - | - | 0.477 |
| **Model 2 + adherence** | - | - | 0.704 |
| **Model 3 + age，sex，BMI** | - | - | 0.548 |
| **Model 4 + AHI** | - | - | 0.548 |

AHI: apnea hypopnea index; BMI: body mass index; e-LFC_NB_: elevated low frequency coupling, narrow-band; PFN-TOTL: Pathfinder Number Test-Total Time

| **Table 6s: Logistic regression model for SWMT-OMD increase after 2 months and 6 months sham-CPAP treatment** | | | |
| --- | --- | --- | --- |
| **2 months after sham-CPAP** | **SWMT-OMD change > 0.65** | | |
|  | OR | 95% CI | p |
| **Model 1: e-LFC_NB_ > 2.35%** | - | - | 0.770 |
| **Model 1 + study site + baseline SWMT-OMD** | - | - | 0.912 |
| **Model 2 + adherence** | - | - | 0.857 |
| **Model 3 + age，sex，BMI** | - | - | 0.857 |
| **Model 4 + AHI** | - | - | 0.857 |
| **6 months after sham-CPAP** | **SWMT-OMD change > 0.8** | | |
|  | OR | 95% CI | p |
| **Model 1: e-LFC_NB_ > 9.45%** | - | - | 0.444 |
| **Model 1 + study site + baseline SWMT-OMD** | - | - | 0.370 |
| **Model 2 + adherence** | - | - | 0.263 |
| **Model 3 + age，sex，BMI** | - | - | 0.219 |
| **Model 4 + AHI** | - | - | 0.219 |

AHI: apnea hypopnea index; BMI: body mass index; e-LFC_NB_: elevated low frequency coupling, narrow-band; SWMT-OMD: Sustained Working Memory Test-Overall Mid-Day Index

| **Table 7s: Logistic regression model for BSRT-SR increase after 2 months and 6 months sham-CPAP treatment** | | | |
| --- | --- | --- | --- |
| **2 months after sham-CPAP** | **BSRT-SR change > 12** | | |
|  | OR | 95% CI | p |
| **Model 1: e-LFC_NB_ > 3.65%** | 1.853 | 0.919-3.736 | 0.085 |
| **Model 1 + study site + baseline BSRT-SR** | 2.267 | 0.918-5.598 | 0.076 |
| **Model 2 + adherence** | - | - | 0.238 |
| **Model 3 + age，sex，BMI** | - | - | 0.381 |
| **Model 4 + AHI** | - | - | 0.381 |
| **6 months after sham-CPAP** | **BSRT-SR change > 4** | | |
|  | OR | 95% CI | p |
| **Model 1: e-LFC_NB_ > 2.15%** | - | - | 0.881 |
| **Model 1 + study site + baseline BSRT-SR** | - | - | 0.923 |
| **Model 2 + adherence** | - | - | 0.679 |
| **Model 3 + age，sex，BMI** | - | - | 0.884 |
| **Model 4 + AHI** | - | - | 0.587 |

AHI: apnea hypopnea index; BMI: body mass index; BSRT-SR: Buschke Selective Reminding Test-Sum Recall; e-LFC_NB_: elevated low frequency coupling, narrow-band

| **Table 8s: Logistic regression model for PFN-TOTL increase after 2 months and 6 months sham-CPAP treatment** | | | |
| --- | --- | --- | --- |
| **2 months after sham-CPAP** | **PFN-TOTL change > -5.8** | | |
|  | OR | 95% CI | p |
| **Model 1: e-LFC_NB_ > 2.35%** | 2.250 | 1.146-4.419 | 0.019 |
| **Model 1 + study site + baseline PFN-TOTL** | 2.413 | 0.970-6.004 | 0.058 |
| **Model 2 + adherence** | - | - | 0.207 |
| **Model 3+age，sex，BMI** | - | - | 0.207 |
| **Model 4 + AHI** | - | - | 0.207 |
| **6 months after sham-CPAP** | **PFN-TOTL change > -2.3** | | |
|  | OR | 95% CI | p |
| **Model 1: e-LFC_NB_ > 2.45%** | - | - | 0.377 |
| **Model 1 + study site + baseline PFN-TOTL** | - | - | 0.148 |
| **Model 2 + adherence** | - | - | 0.373 |
| **Model 3 + age，sex，BMI** | - | - | 0.627 |
| **Model 4 + AHI** | - | - | 0.627 |

AHI: apnea hypopnea index; BMI: body mass index; e-LFC_NB_: elevated low frequency coupling, narrow-band; PFN-TOTL: Pathfinder Number Test-Total Time
